# Navigating anticoagulation in heart transplantation with thrombin–antithrombin complex: when conventional monitoring fails

**DOI:** 10.1186/s13019-026-04500-3

**Published:** 2026-07-28

**Authors:** Chaonan Liu, Liqin Ling, Xunbei Huang, Zhiyu Yu, Shuang Wang, Jing Zhou

**Affiliations:** 1 Department of Laboratory Medicine, West China Hospital, Sichuan University, Chengdu, China; 2 Clinical Laboratory Medicine Research Center of West China Hospital, Sichuan Clinical Research Center for Laboratory Medicine, Sichuan University, Chengdu, China

**Keywords:** Heart transplantation, Thrombin-antithrombin complex, Anticoagulation management, Postoperative thrombosis

## Abstract

**Background:**

Heart transplant recipients often face the clinical dilemma of coexisting bleeding and thrombotic risks postoperatively. Conventional anticoagulation monitoring parameters (e.g., platelet count, fibrinogen, D‑dimer, and anti‑Xa activity) frequently exhibit limitations in evaluating complex postoperative patients, including delayed response, poor sensitivity, and lack of clear target ranges. This case report describes the use of thrombin–antithrombin complex (TAT) to guide anticoagulation management for complex thrombotic events after heart transplantation and explores its potential value as a supplementary monitoring tool.

**Case presentation:**

A 50‑year‑old male heart transplant recipient developed multiple venous thrombi in the extremities and cerebral venous sinuses during the early postoperative period. Conventional monitoring parameters (platelets, fibrinogen, D‑dimer, and anti‑Xa activity) failed to effectively warn of thrombus progression or guide dose adjustments. Thrombosis continued to progress despite increasing anticoagulant intensity. Introduction of TAT monitoring revealed that its dynamic changes closely paralleled thrombotic events. Persistently elevated TAT levels (> 8 ng/mL) indicated inadequate anticoagulation. After adjusting anticoagulant intensity based on TAT levels to maintain them within a safe range (< 8 ng/mL), thrombosis was controlled. Later, when anti‑Xa activity decreased due to infection, stable TAT levels provided evidence to maintain the original anticoagulation regimen, avoiding overtreatment.

**Conclusions:**

This case suggests that TAT may serve as a useful adjunctive monitoring tool for hypercoagulable states after heart transplantation. Its dynamic changes may more sensitively reflect thrombotic risk and assist in clinical decision‑making, particularly when conventional parameters are contradictory or lack clear targets, thereby facilitating more individualized and precise anticoagulation management.

**Supplementary Information:**

The online version contains supplementary material available at https://doi.org/10.1186/s13019-026-04500-3.

## Background

Heart transplant patients are at high risk of perioperative bleeding; therefore, the International Society for Heart and Lung Transplantation (ISHLT) [1] recommends considering anticoagulation only in specific clinical scenarios, such as postoperative atrial or ventricular arrhythmias, to reduce graft intravascular thrombosis associated with antibody‑mediated acute rejection, and during mechanical circulatory support with devices like Extracorporeal Membrane Oxygenation (ECMO). Current guidelines clearly recommend initiating anticoagulants (including direct oral anticoagulants or vitamin K antagonists) for post‑transplant atrial fibrillation/flutter based on the CHADS‑VASc score. However, existing guidelines still lack specific operational guidance on monitoring anticoagulation efficacy and balancing thrombotic and bleeding risks. Thrombin–antithrombin complex (TAT) [2] is a direct molecular marker of thrombin generation, which may reflect the activation of the coagulation system earlier and more sensitively than conventional markers. This case report explores the use of TAT to evaluate anticoagulation efficacy in a heart transplant recipient, aiming to help guide effective anticoagulation while minimizing bleeding risk.

## Case presentation

A 50‑year‑old male with ischemic cardiomyopathy, coronary artery atherosclerosis, acute exacerbation of chronic heart failure (NYHA class IV), and arrhythmias was scheduled for heart transplantation at our institution. His medical history included hypertension for 30 years, coronary heart disease for 18 years (coronary stenting 10 years prior), chronic hepatitis B virus infection for 8 years, type 2 diabetes for 4 years, cerebral infarction 2 years prior, and hyperuricemia for 1 year. Preoperative medications included atorvastatin, rivaroxaban, clopidogrel, furosemide, spironolactone, digoxin, sacubitril valsartan, bisoprolol, and empagliflozin. In October 2025, he underwent “allogeneic heart transplantation and placement of temporary epicardial pacing wires” and was transferred to the intensive care unit. Laboratory tests revealed mild anemia, slightly low platelets, and markedly elevated infection markers (detailed in *Supplementary Table 1*). Treatment included mycophenolate mofetil, tacrolimus, methylprednisolone sodium succinate, esomeprazole, piperacillin‑tazobactam, ambroxol hydrochloride, febuxostat, ambrisentan, spironolactone, and atorvastatin calcium for supportive care.

**Fig. 1:**
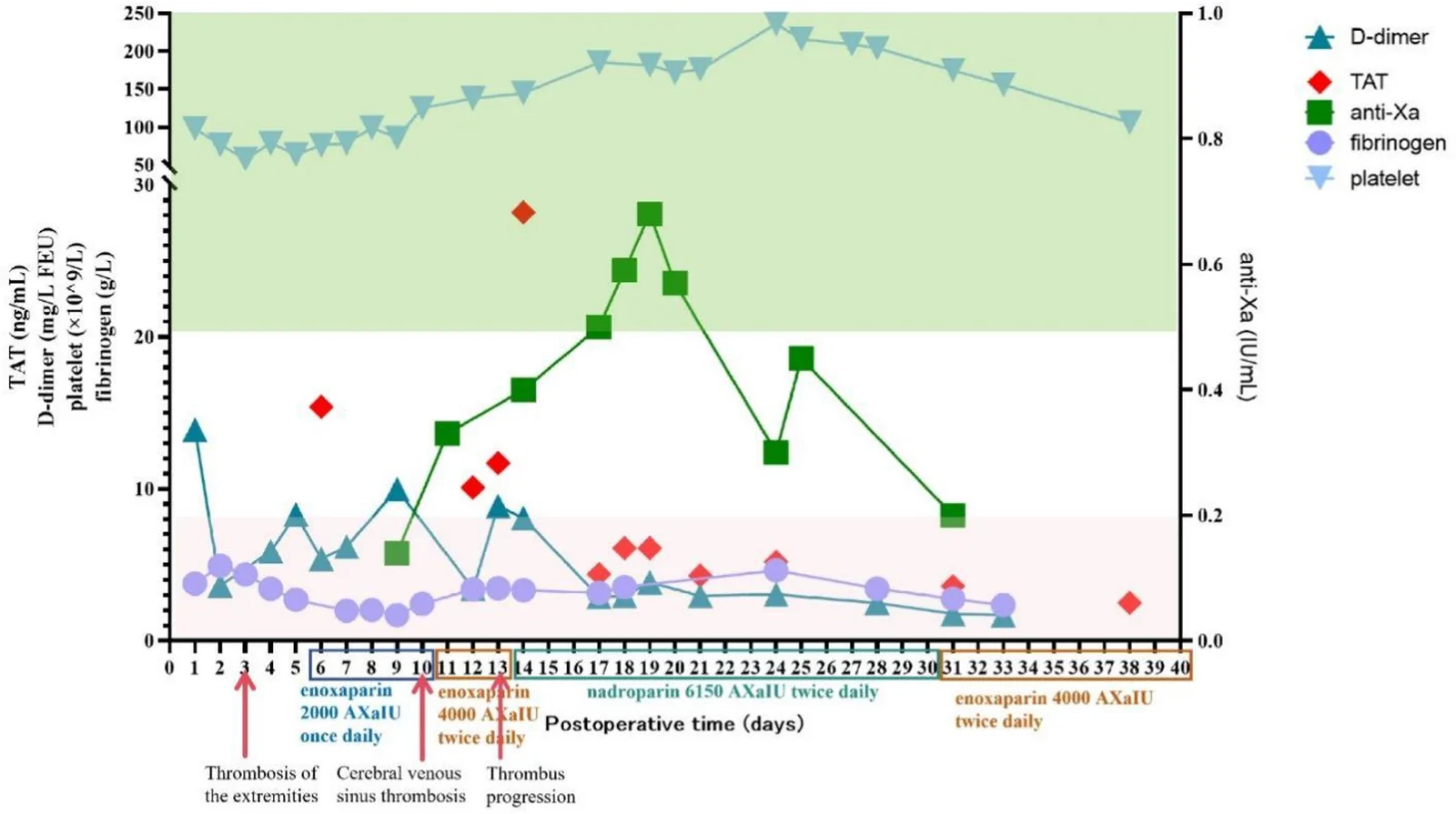
Dynamic changes in thrombin–antithrombin complex (TAT) and conventional coagulation parameters in relation to thrombotic events and anticoagulation adjustment. Dynamic changes in D-dimer (dark blue), fibrinogen (purple), platelet count (light blue), anti‑Xa activity (green), and thrombin–antithrombin complex (TAT, red) from postoperative day 1 to day 40. TAT values are plotted without connecting lines due to intermittent monitoring. The green shaded area indicates the recommended target anti‑Xa range (0.5–1.0 IU/mL) for non‑surgical patients. The pink shaded area represents the proposed safe threshold for TAT (< 8 ng/mL). The left vertical axis corresponds to D‑dimer, fibrinogen, platelet count, and TAT; the right vertical axis refers to anti‑Xa activity. Horizontal bars above the timeline denote the dosing periods of low‑molecular‑weight heparin. Red arrows indicate time points at which thrombosis was detected or showed progression

On postoperative day (POD) 3, the patient developed multiple thrombotic events: vascular ultrasound of the extremities indicated thrombosis in the right cephalic vein, left superficial femoral vein, popliteal vein, and posterior tibial vein, as well as ischemia in the mid‑upper segment of the right superficial femoral artery, the origin of the left posterior tibial and anterior tibial arteries, and the left radial artery. Laboratory work‑up ruled out typical antiphospholipid antibody syndrome (lupus anticoagulant, anti‑cardiolipin antibody, and anti‑β2GP1 antibody all negative) and thrombotic thrombocytopenic purpura (PLASMIC score 4 [3], with lactate dehydrogenase and indirect bilirubin steadily declining from postoperative peaks). The main dilemma was: the patient had a low platelet count (58 × 10⁹/L) (Fig. 1), a MELD‑XI score of 12.7, and high early postoperative bleeding risk; however, imaging had confirmed multiple thrombi, necessitating anticoagulation. Thromboelastography showed no significant abnormalities in coagulation factors, fibrinogen, platelet function, or the fibrinolytic system. After thorough evaluation and transfusion of one therapeutic dose of platelets, cautious prophylactic anticoagulation was initiated with enoxaparin 2000 AXaIU once daily, considering the high bleeding risk.

On POD 5, the patient developed involuntary jaw twitching and increased lower limb muscle tone, with negative pathological reflexes. Cranial computed tomography (CT) revealed a right frontal lobe cerebral infarction with encephalomalacia, a few ischemic foci intracranially, normal ventricular and cisternal morphology, and no midline shift. By POD 7, lower limb muscle tone further increased, intermittently presenting as forced flexion. Repeat cranial CT showed no significant changes. On POD 10, cranial magnetic resonance imaging (MRI) clearly identified multiple thrombi within the venous sinuses (right sigmoid sinus, bilateral transverse sinuses, superior sagittal sinus, and confluence of sinuses); a right frontal periventricular encephalomalacia with gliosis; and white matter hyperintensities (Fazekas grade 2), considered ischemic or demyelinating lesions. Given that haloperidol had been initiated on POD 3 for agitation, the subsequent neuropsychiatric symptoms were considered possibly drug-related. Haloperidol was therefore discontinued, after which no further dystonia occurred. In light of the serious complication of cerebral venous sinus thrombosis, the anticoagulation regimen was intensified to enoxaparin 4000 AXaIU twice daily. Anti‑Xa activity measured 4 h after the first dose of this regimen was 0.33 IU/mL. The core dilemma here was: there is no recommended anti‑Xa target range for anticoagulation after heart transplantation. The widely cited standard range of 0.5–1.0 IU/mL [4] originates from non‑major‑surgery patients; applying it directly to this high‑bleeding‑risk patient could increase danger. Furthermore, anti‑Xa activity requires at least 3 doses to reach steady state, so the dose was not immediately increased further. After completing 3 doses, repeat anti‑Xa was 0.4 IU/mL.

Despite intensified anticoagulation, thrombosis progressed. Repeat extremity venous ultrasound on POD 13 showed new thrombi in the right internal jugular vein, around the left brachial vein catheter, and in bilateral calf muscular veins; cranial MRI also revealed a new thrombus in the left sigmoid sinus. At this point, conventional monitoring parameters reached an impasse: anti‑Xa activity (0.4 IU/mL) had not reached the lower limit of the conventional therapeutic range for non‑surgical patients (0.5 IU/mL); platelet count and fibrinogen showed no persistent consumption; D‑dimer levels, although fluctuatingly elevated (from 5.88 to 9.98 mg/L FEU), lacked a clear correlation with thrombotic events. In subsequent monitoring, even when anti‑Xa reached 0.5 IU/mL, D‑dimer remained elevated at 2.9 mg/L FEU (5‑fold above the upper limit of normal), with a transient rise to 3.84 mg/L FEU without acute thrombotic evidence. This contradictory state made clinical decision‑making extremely difficult: rapidly escalating anticoagulant intensity to the conventional target range based on guidelines [4] could pose an unacceptable bleeding risk; maintaining the current regimen based on the non‑specific changes in platelets, fibrinogen, and D‑dimer might allow life‑threatening (especially to the graft heart) thrombosis to progress.

At this critical decision point, we reviewed and analyzed the already measured TAT levels. The results were highly suggestive: on POD 6, TAT was already elevated at 15.4 ng/mL; even after initiating low‑intensity anticoagulation, TAT remained at 10.1 ng/mL on POD 12. On POD 13, when imaging confirmed thrombus progression, TAT levels were 11.7 ng/mL and subsequently 28.2 ng/mL on POD 14. This series of data consistently indicated a persistent and worsening hypercoagulable state. Based on this finding, and considering our previous research [5] suggesting that maintaining TAT below 8 ng/mL is associated with a significantly reduced thrombotic risk, we made an exploratory decision: to increase anticoagulant intensity by switching to nadroparin 6150 AXaIU twice daily to minimize waste, aiming to rapidly suppress TAT levels below the safety threshold. After intensification, the effect was significant: TAT levels promptly decreased to 4.4 ng/mL and subsequently stabilized within the safe range of 4.3–6.1 ng/mL; concurrently, anti‑Xa activity stabilized at 0.5–0.68 IU/mL. Imaging assessment showed halted thrombus progression, and repeat extremity venous ultrasound on POD 21 indicated reduced thrombus burden. This preliminarily validated the effectiveness of TAT‑guided anticoagulation adjustment in this case.

A more complex situation arose later in the postoperative course. On POD 24, without any change in the anticoagulant dose, the patient’s anti‑Xa activity unexpectedly dropped sharply from the previous 0.5–0.68 IU/mL to 0.3 IU/mL. Of note, antithrombin (AT) activity was normal (98.0%) at this time point (Supplementary Table 1). According to conventional anticoagulation monitoring logic, this change suggested potentially insufficient anticoagulant effect and a theoretical risk of thrombosis recurrence, typically warranting consideration of intensification. However, the concurrent TAT level was stable at 5.2 ng/mL (below the 8 ng/mL safety threshold). At this time, respiratory nucleic acid testing newly identified a mixed infection with Klebsiella pneumoniae, Pseudomonas aeruginosa, and Acinetobacter baumannii. Severe infection is known to elevate multiple acute-phase proteins, including heparin‑binding protein [6], vitronectin, and α1-acid glycoprotein, which may bind and neutralise heparin (including LMWH), thereby contributing to reduced anti‑Xa activity [7]. The stable TAT level provided important evidence that coagulation activation remained effectively suppressed. Therefore, we made a decision contrary to conventional logic: we did not increase the anticoagulant dose based solely on the isolated, potentially confounded drop in anti‑Xa activity. The subsequent clinical course supported this decision: the patient developed no new thrombi thereafter.

This patient had significantly elevated procalcitonin early postoperatively. Subsequent sputum culture and pathogen metagenomic sequencing identified Candida albicans, Staphylococcus aureus, and multidrug‑resistant Gram‑negative bacterial infections, indicating that infection was an important underlying etiology triggering and sustaining the hypercoagulable state. As targeted anti‑infective therapy (including caspofungin, vancomycin, meropenem, colistin, etc.) took effect, infection markers gradually normalized. After effective infection control, we attempted to de‑escalate the anticoagulation regimen to enoxaparin 4000 AXaIU twice daily on POD 31 for de-escalation purposes and waste minimization, with TAT monitoring confirming sustained efficacy. At this time, monitored anti‑Xa activity was only 0.2 IU/mL, but TAT remained at a low level of 3.6 ng/mL. The patient developed no new thrombi under this regimen and was discharged smoothly on POD 45. Post‑discharge, anticoagulation was switched to rivaroxaban 10 mg once daily orally. Three‑month outpatient follow‑up revealed no thrombotic events.

## Discussion and conclusions

This case details the management of a heart transplant recipient who developed rare, complex, and recurrent thrombosis postoperatively. The uniqueness lies not only in the extensiveness of thrombotic events (involving peripheral arteries/veins and cerebral venous sinuses) but also in the clear limitations exposed by conventional anticoagulation monitoring tools in this setting. First, anti‑Xa activity lacked an established standard. There is a lack of specific anticoagulation targets for the post‑heart‑transplant population. The applicability of the generic 0.5–1.0 IU/mL range to these high‑bleeding‑risk patients remains unclear. In this case, on POD 13 when thrombosis progressed, anti‑Xa was only 0.4 IU/mL, neither reaching the generic target nor providing a definitive basis for dose adjustment in this context. Second, traditional parameters provided feedback that was often delayed or inconsistent regarding clinical events. The non‑specific fluctuations of indicators like D‑dimer limited their utility as reliable real‑time guides for dose adjustment.

Within the “blind spot” and decision-making dilemma of conventional monitoring, dynamic TAT monitoring provided useful information. In this case, changes in TAT levels closely synchronized with radiological evidence of thrombus progression. More importantly, it provided evidence at two critical decision-making junctures: First, when traditional indicators (anti‑Xa 0.4 IU/mL) offered no clear guidance, the significant elevation of TAT (up to 28.2 ng/mL) suggested inadequate anticoagulation, prompting us to undertake prompt dose intensification. Second, when anti‑Xa activity unexpectedly decreased (to 0.3 IU/mL) suggesting theoretical risk despite unchanged anticoagulant intensity, the stable TAT level (5.2 ng/mL) supported maintaining the current regimen, thereby avoiding unnecessary dose escalation and potential bleeding risks. These two contrasting scenarios highlight the potential of TAT to assist in decision‑making in complex clinical situations.

This exploratory practice suggests that incorporating TAT into the anticoagulation monitoring framework for high‑risk, complex patients such as those after heart transplantation may enable more individualized management. Its key advantage lies in TAT being a direct marker of thrombin generation, thereby potentially better accurately reflecting in vivo coagulation activity. Based on previous research and observations from this case, we tentatively propose maintaining TAT below 8 ng/mL as a potential safety management target, which provided a useful reference for the successful management of this patient.

Several limitations should be acknowledged. First, TAT immunoassays are not yet universally available. In our hospital, TAT is a routine test (instrument run time ~ 20 min, total turnaround time around 1–4 h) using a fully automated chemiluminescence immunoassay (HISCL‑5000, Sysmex), which supports real‑time clinical decisions; however, this level of accessibility may not be generalizable to other settings. Second, the proposed safety threshold (TAT < 8 ng/mL) and the optimal monitoring frequency require prospective validation in larger cohorts of heart transplant recipients. Third, as a single case report, causal inference and generalizability are inherently limited. The discharge anticoagulation regimen was an individualised exploration, so caution is needed when applying this approach to other settings. Finally, TAT should be considered a supplementary tool to conventional monitoring rather than a replacement; while it helps assess the adequacy of anticoagulation, it does not directly evaluate bleeding risk. Therefore, clinical decisions must always integrate multiple laboratory parameters and judgement.

In this case, conventional anticoagulation monitoring provided limited real‑time guidance, whereas serial TAT dynamically reflected coagulation activation and assisted decision‑making at critical junctures. Although TAT is not yet universally available and requires further validation, this case suggests that incorporating TAT into the monitoring framework may enable more individualized anticoagulation management in complex post‑transplant patients.

## Supplementary Information

Below is the link to the electronic supplementary material.

Supplementary Material 1: Table 1. Key laboratory and clinical course data of the presented case.

## Data Availability

The authors confirm that the data supporting the findings of this study are available within the article.

## Abbreviations

TAT: Thrombin–antithrombin complex
POD: Postoperative day
