# Supplementary material for: Navigating anticoagulation in heart transplantation with thrombin–antithrombin complex: when conventional monitoring fails
Source: J Cardiothorac Surg. 2026 Jul 28;21:840. doi: 10.1186/s13019-026-04500-3 (PMC13693143; doi:10.1186/s13019-026-04500-3)
Supplement: Supplementary file 1 [file 13019_2026_4500_MOESM1_ESM.docx]

Supplementary Table 1. Key Laboratory and Clinical Course Data of the Presented Case.

|  | POD  -1 | POD 1 | POD 2 | POD  3 | POD  4 | POD 5 | POD 6 | POD 8 | 9 Day | 10 Day | 11 Day | 12 Day | 13 Day | 14 Day | 17 Day | 18 Day | 19 Day | 20 Day | 21 Day | 24 Day | 28 Day | 31 Day |
| --- | --- | --- | --- | --- | --- | --- | --- | --- | --- | --- | --- | --- | --- | --- | --- | --- | --- | --- | --- | --- | --- | --- |
| PLT, 10^9/L | 174 | 98 | 77 | 58 | 79 | 64 | 76 | 99 | 86 | 125 |  | 138 |  | 145 | 185 |  | 182 | 172 | 176 | 236 | 204 | 175 |
| HGB, g/L | 140 | 99 | 100 | 98 | 100 | 95 | 98 | 101 | 109 | 100 |  | 86 |  | 93 | 87 |  | 99 | 97 | 93 | 107 | 104 | 103 |
| RBC, 10^12/L | 4.76 | 3.3 | 3.34 | 3.26 | 3.39 | 3.23 | 3.27 | 3.48 | 3.73 | 3.35 |  | 2.87 |  | 3.2 | 3.05 |  | 3.37 | 3.33 | 3.14 | 3.72 | 3.59 | 3.6 |
| WBC, 10^9/L | 5.8 | 12.98 | 16.69 | 9.21 | 7.41 | 5.23 | 4.62 | 5.94 | 7.13 | 10.54 |  | 6.45 |  | 5.63 | 5.56 |  | 6.29 | 6.6 | 6.25 | 5.73 | 4.79 | 4.92 |
| Neu, % | 59.2 | 90.9 | 92.6 | 95.2 | 95.9 | 92.7 | 84.4 | 85.5 | 84.5 | 86.9 |  | 89.2 |  | 83.2 | 86.5 |  | 79.8 | 87.2 | 81.7 | 72.6 | 71.9 | 67.9 |
| PT, s | 23.8 | 13.7 | 17.4 | 16.1 | 14.9 | 15.5 | 14.8 | 12 | 11.6 | 11 |  | 10.6 | 10.1 | 10.4 | 10.6 |  | 10.5 |  | 10.9 | 10.9 | 10.8 | 10.5 |
| APTT, s | 28.8 | 25.4 | 15.7 | 28.2 | 26 | 26.1 |  | 25 | 23.7 | 21.4 |  | 25.1 | 25.9 | 21.7 | 26.7 |  | 28.5 |  | 28.9 | 27.3 | 28.2 | 29.6 |
| FIB, g/L | 4.16 | 3.77 | 4.94 | 4.37 | 3.43 | 2.71 |  | 2.04 | 1.70 | 2.43 |  | 3.39 | 3.46 | 3.33 | 3.15 | 3.53 |  |  |  | 4.65 | 3.43 | 2.76 |
| AT, % |  | 74.2 | 76.4 |  | 94.1 |  |  |  | 96.2 |  |  | 101.4 | 103.7 | 116.7 | 105.5 |  |  |  | 88.3 | 98.0 | 92.7 | 100.5 |
| D-dimer, mg/L FEU |  | 13.89 | 3.63 |  | 5.88 | 8.30 | 5.40 |  | 9.98 |  |  | 3.43 | 8.86 | 8.08 | 2.90 | 2.95 | 3.84 |  | 2.95 | 3.06 | 2.5 | 1.77 |
| TAT, ng/mL |  |  |  |  |  |  | 15.40 |  |  |  |  | 10.10 | 11.70 | 28.20 | 4.40 | 6.10 | 6.10 |  | 4.30 | 5.20 |  | 3.6 |
| Anti-Xa, IU/mL |  |  |  |  |  |  |  |  | 0.14 |  | 0.33 |  |  | 0.40 | 0.50 | 0.59 | 0.68 | 0.57 |  | 0.30 |  | 0.2 |
| LMWH |  |  |  |  |  |  | 2000 AXaIU once daily | | | | 4000 AXaIU twice daily | | | 6150 AXaIU twice daily | | | | | | | | 4000 AXaIU twice daily |
| Thrombosis |  |  |  | Thrombosis of the extremities |  |  |  |  |  | Cerebral venous sinus thrombosis |  |  | Thrombus progression |  |  |  |  |  |  |  |  |  |

PLT, platelet. HGB, hemoglobin. RBC, erythrocyte. WBC, leukocyte. Neu, neutrophil. PT, prothrombin time. APTT, activated partial thromboplastin time. FIB, fibrinogen. TAT, thrombin-antithrombin complex. LMWH, low molecular weight heparin. POD, postoperative Day.
